# Development of a Scoring Tool for Australian Rural Food Retail Environments

**DOI:** 10.3390/nu15214660

**Published:** 2023-11-03

**Authors:** Tracy L. Schumacher, Carissa A. Alderton, Leanne J. Brown, Susan Heaney, Laura Alston, Katherine Kent, Stephanie Louise Godrich

**Affiliations:** 1 Department of Rural Health, College of Health, Medicine and Wellbeing, University of Newcastle, Tamworth, NSW 2340, Australia; carissa.alderton@newcastle.edu.au (C.A.A.); leanne.brown@newcastle.edu.au (L.J.B.); 2 Food and Nutrition Research Program, Hunter Medical Research Institute, New Lambton, NSW 2305, Australia; susan.heaney@newcastle.edu.au; 3 Department of Rural Health, College of Health, Medicine and Wellbeing, University of Newcastle, Port Macquarie, NSW 2444, Australia; 4 Deakin Rural Health, School of Medicine, Deakin University, Geelong, VIC 3220, Australia; laura.alston@deakin.edu.au; 5 Research Unit, Colac Area Health, Colac, VIC 3250, Australia; 6 School of Medical, Indigenous and Health Sciences, University of Wollongong, Wollongong, NSW 2522, Australia; katherinek@uow.edu.au; 7 School of Health Sciences, University of Tasmania, Launceston, TAS 7250, Australia; 8 Centre for People, Place, and Planet, Nutrition and Health Innovation Research Institute, School of Medical and Health Sciences, Edith Cowan University, Joondalup, WA 6027, Australia; s.godrich@ecu.edu.au

**Keywords:** rural, food environment, food retail, diet, nutrition

## Abstract

Current tools scoring the healthiness of food retail outlets do not reflect outlets found in rural locations. This study aimed to adapt pre-existing Australian scoring tools to represent non-metropolitan areas. Rural nutrition experts were identified, and a modified Delphi technique was used to adapt two pre-existing, food-scoring tools in five iterative stages. Stages included identifying all relevant outlets, providing a description and score for each, ensuring consistency between outlet scores and pre-existing, metro-centric tools, and providing instructions for correct use. Six rural nutrition experts were identified and engaged in the modified Delphi technique. The final tool consisted of 12 categories of food outlets and listed 35 individual outlets. Consistent with pre-existing Australian tools, scores ranged from +10 to −10 and included descriptions reflective of rural retail outlets. Scores were based on whether the majority of foods offered within the outlet were consistent with foods recommended in national health guidelines. The developed tool was designed to accommodate the diverse nature of food retail outlets found in non-metropolitan areas. This study assists in explaining the link between the food environment and health in populations living rurally.

## 1. Introduction

Australian populations, like those in other high-income countries, do not—or are unable—to adhere to a diet that is consistent with national dietary guidelines [1,2,3]. For example, the most recent national data from the 2020–2021 National Health Survey show that less than half of adults consume the recommended servings of fruit, and less than 1 in 10 adults consume the recommended servings of vegetables [4]. Poor dietary patterns, including low intake of fruits and vegetables, have been identified as a large contributor to the high burden of chronic diseases globally [5]. The role of dietary intake on rural health inequities is well evidenced and ongoing [6]. While individual-level factors such as food knowledge, beliefs, and habits have been established as predictors of dietary outcomes [7], there is also a growing body of evidence suggesting that the food environment plays a significant role in shaping dietary behaviours [8]. The food environment refers to the physical, economic, social, and cultural factors that influence an individual’s access to, availability of, and choice of food. The physical characteristics of food environments such as the availability of food and access to food, in addition to food marketing and advertising and socioeconomic factors, are purported to be major drivers of less healthy population-level dietary intakes. In particular, the type, location, healthiness, and number of food outlets within a geographic location have been recognized as the environmental determinants likely to have the greatest impact on population diets and subsequent outcomes of health [9]. Evidence suggests that dietary intake is a key driver of health inequalities, which may be influenced by differences in the food environments in these locations [10].

The current evidence of the influence of food environments in Australia on dietary intake and health outcomes is limited due to a lack of data beyond Melbourne, Victoria, where around two-thirds of studies have been conducted [11]. In addition, a recent review has suggested inherent limitations and heterogeneity in the methods applied across the literature, which could explain the mostly inconclusive and conflicting findings [11]. To build a more robust body of evidence that informs policies and planning initiatives to positively impact food environments across Australia, more studies on the location, accessibility, and healthiness of food outlets across more diverse regions of Australia are warranted. In particular, a focus on populations that are most affected by issues with food access and supply, such as rural and regional areas, is required through accurate mapping of the food environments.

In some studies from the urban regions of Australia, healthier food environments have been associated with better diet quality and food security [12]. However, the pathways through which food environments influence dietary intake and food insecurity in the regional areas of Australia remain under-researched [13], along with a lack of broader nutrition research in rural areas [14]. Preliminary evidence suggests that some regional food environments are less healthy and lack the promotion of healthy foods [12,15]. However, this may be due to pre-existing “metro-centric” tools for measuring food environments, which have been argued to be invalid in non-urban settings for several reasons [12]. Firstly, the availability and accessibility of food outlets and types of food establishments (and their healthiness) vary, and evidence suggests that dietary preferences and patterns differ in non-urban areas [16]. For example, rural areas often have a higher prevalence of combination outlets, which encompass general stores, bottle shops, and take-away establishments. Additionally, there is considerable variation in the types of food available in rural outlets, with a tendency towards more shelf-stable foods, such as processed items in general stores where groceries can be found. Secondly, the cultural and socio-economic context of non-urban areas can significantly impact the food environment. For example, local food production, agricultural practices, and community engagement with food systems may differ, such as more localised food systems, requiring specific considerations when assessing food environments in these settings. A 2019 review by Love et al. [12] found 25 studies that sought to measure the food environment across non-metropolitan Australia and found that pre-existing tools were often metro-centric, not contextual to rural areas, and not validated in rural settings. The review identified a need for more consistent and relevant tools to measure rural food environments.

Existing tools for measuring food environments in Australia are unable to effectively capture these distinct characteristics of the food environment in rural areas, in particular, the prevalence of combination outlets (general store/bottle shop/take away) and the variation in the types of food available. More specifically, the tools may also underestimate alcohol access by not measuring drive-thru bottle shops attached to hotels or general stores. Consequently, these tools may not accurately represent the rural food environment across Australia, highlighting the need for more comprehensive approaches tailored to rural settings. Therefore, the aim of this project was to adapt existing Australian food environment healthiness scoring tools to better describe food outlets commonly found in rural communities (classified as Modified Monash Model remoteness areas 3–5) [17] using a modified Delphi technique with rural nutrition experts [18].

## 2. Materials and Methods

### 2.1. Setting

This tool aims to be used in areas in Australia categorised as 3–5 using the Modified Monash Model system [17], which is based on the Australian Statistical Geography Standard (ASGS) and ASGS of Remote Areas (ASGS-RAs) [19,20]. Areas designated MM3–5 must be categorised as ASGS-RA2 (Outer Regional Australia) or ASGS-RA3 (Remote Australia) [19]. Large rural towns (MM3) are areas within 15 km of road distance of a town with a population of 15–50,000 people. Medium rural towns (MM4) include towns that are within 10km of a town of 5000–15,000 people. Small rural towns (MM5) are all other towns listed in ASGS-RA2 or RA3 and are not categorised as MM1–4.

### 2.2. Development of the Scoring Tool

#### 2.2.1. Population

Rural academics from across Australia with expertise in either nutrition or dietetics were approached to participate via email. Academics invited to participate in the development of the scoring tool needed to: (1) hold PhD qualifications; (2) demonstrate expertise about the food environment in their respective rural area; (3) have at least one publication relating to food environments in a rural area; (4) have personal experience of food environments in geographical areas designated as MM3–5 by the Modified Monash Model [17]. Group members were identified via formal and informal rural research networks, and the snowball method was additionally used to extend networks. These nutrition experts were expected to present and critique information that related to their own personal experience of their rural area. Ethics was not sought for this collaborative project, as all involved personnel were considered contributing authors due to the extent of expert opinion that was expected in all stages of the development of the tool.

#### 2.2.2. Process

**Figure 1:**
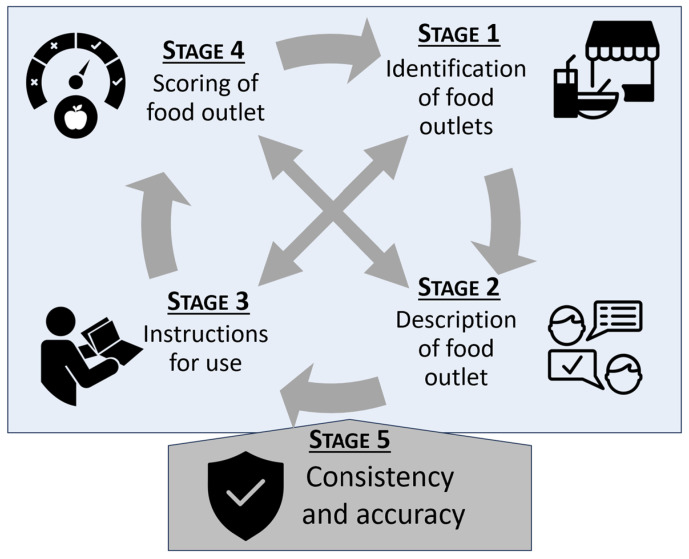
Depiction of process undertaken using a modified Delphi technique.

The process of adapting previous tools from the literature occurred in five iterative and overlapping stages and was guided by previous literature [21,22]. All work was considered in its entirety and could be modified at any time to reach a consensus with the group. Group consensus was pre-defined as all members responding as neutral, in agreement or in strong agreement with proposed statements and/or scores, as opposed to some or all members identifying as in disagreement or strong disagreement. Consensus could be removed from previous stages if a member no longer agreed with statements and/or scores. A research assistant was appointed to guide the first stages of the process and to provide anonymity as much as possible. Stage 1 identified rural food outlets of interest. Stage 2 provided descriptions for each of the outlets. Stage 3 focused on developing instructions for use that described how the tool was to be used. Stage 4 occurred concurrently with Stage 3 and aimed to provide a healthiness score for each type of outlet that was compatible with other scores provided in the literature. Stage 5 was to ensure consistency within the tool. Importantly, a consensus also had to be reached from the expert group that this tool could be used in their individual areas of expertise and that it was an accurate representation (see Figure 1).

#### 2.2.3. Stage 1: Identification of Rural Food Outlets of Interest

Two existing tools (Needham et al. [23] and Moayyad et al. [24]) were identified from the Australian food environment literature that were considered to be suitable for adaptation to the rural context due to the range of outlets included [24] or the descriptions attributed [23]. These tools were presented to the group of experts in an online meeting, where any limitations that might apply to the rural environment were raised and discussed.

All food outlets identified by Needham et al. and Moayyad et al. were presented for potential inclusion [23,24]. Also presented were food outlet types derived from White and Yellow Pages searches (2018/19) [25]. The 2018/19 White and Yellow Pages were used in preference to more recent editions due to the change in marketing for food outlets, in which hardcopy decreased in popularity due to a rise in online advertising in areas affected by COVID-19. White and Yellow Pages had the additional benefit of providing a more comprehensive listing of different outlets in rural regions, compared to using online media. Additionally, suggestions from the expert group that were based on food outlets that existed in their own rural areas of expertise were also considered.

#### 2.2.4. Stage 2: Development and Modification of Outlet Descriptions

A survey was developed in REDCap [26,27] and aimed to capture a level of agreement regarding rural food outlet categories and their descriptions for inclusion in the tool. The anonymous survey was sent to the group of experts and comprised a total of 36 food outlet categories for potential inclusion. The survey consisted of three sub-sections: (A) food outlets with existing descriptions; (B) food outlets without an existing description; (C) food outlets that may fit within an existing category (see Table 1), as well as instructions for each section.

During a series of weekly cycles of surveys followed by online meetings, de-identified and pooled survey agreement results from Part A (food outlets with existing descriptions) were presented to the expert group at the meeting, which was recorded to ensure accurate interpretation. Consensus from “Food outlets with an existing description” was deemed to reach agreement if all participants voted either ‘neutral’, ‘agree’, or ‘strongly agree’ for the proposed description or all participants verbally indicated agreement during a meeting. Modifications made during team meetings were emailed to members immediately after meetings, ready for the next iteration. Types of changes made were identified as follows: (i) modifications made to pre-existing descriptions were added in red text to differentiate between modifications and the original wording; (ii) any pre-existing wording that was removed was indicated using strikethrough; (iii) new categories of retail outlets or where outlets categories were amended were documented in blue text.

Within the iterations of meetings and surveys, the expert group was also asked to propose descriptions for Part B (food outlets without an existing description). The proposed descriptions were held until the majority of Part A had reached an agreement for inclusion. The descriptions were then presented to the expert group in a follow-up online survey. Participants were asked to rank descriptions presented from the survey in order of preference. Pooled ranked preferences were provided to the group and modifications were made during a continuation of online meetings. Weekly meetings were halted once the expert group agreed with the outlets listed and their descriptions for this stage.

Outlet types from Part C not already included were reiteratively addressed in the following stages, and whether to include them as their own description, include them within another pre-existing category, or exclude them from the tool was considered.

#### 2.2.5. Stage 3: Instructions for Use

Instructions for use were developed iteratively and evolved in conjunction with descriptions and scoring. For example, definitions for terms used in the outlet descriptions were required in some instances to ensure consistent interpretation, and to ensure that the tool was used according to intent after completion. Where possible, definitions were obtained from government sources or other established credible sources.

Additionally, as the size of an outlet can have ramifications for the level of access that it offers, a definition from a government source was provided that could be used for categorising an outlet by its size, and, therefore, its reach [28].

#### 2.2.6. Stage 4: Providing a Healthiness Score to Included Outlets with Descriptions

To ensure the consistency of scoring with pre-existing literature, the same scoring range as provided by Moayyed et al. [24] was used and ranged from −10 (very unhealthy) to +10 (very healthy). The outlet list with previously agreed descriptions from Stage 2 was presented via email in an editable document to the group, with a space for independently adding a score that reflected their food environment of expertise. Where a score from the literature already existed, this was provided for context [23,24]. The instructions given to the group considered the terminology agreed on earlier to score each category and sub-category relative to each other and relative to other outlets in the tool.

Returned responses were collated and the document was then re-circulated, this time including the score from the literature, the individual answers from the expert group (random ordering), the mean and standard deviation of the submitted answers, and space for a new answer. This process was undertaken so that the stability of the answers was able to be identified. Large discrepancies in answers, indicating a possible lack of stability, were likely due to either a different interpretation provided by the description or because different foods were offered in the outlet described. Where this occurred, the description was revisited to ensure that either the interpretation was clear or that an option was available where different foods were offered by that outlet.

Additionally, after the first round of individual scoring was performed, a visual scale of existing answers was constructed to compare the relativity of each outlet score more easily. Each outlet was positioned according to the mean score of the group, with the standard deviation indicating how far from disagreement the group was. Any outlets with a standard deviation of 0 were colour-coded as a consensus was reached. A guide was provided to the expert group that all other outlets may be moved to the extent of the standard deviation of the individual scores. For example, an outlet with a mean of +3 and a standard deviation of 5 could be moved up to five increments in either direction.

The editable document and visual scale were presented to the group for additional rounds of individual scoring. Answers were again collated. The process was to continue until either consensus was complete or when no further progress was made with discrepancies.

#### 2.2.7. Stage 5: Consistency and Consensus

Experts were sent an editable document and two visual scales depicting the current food-scoring outlet tool as a whole so that the scoring could be seen in its entirety and allow for a comparison between categories. One visual scale was presented as the current mean score, and the other was where it was rounded to the nearest whole number, with the actual mean also provided. Members were asked to consider the visual scale of the tool and to identify any outlets considered to be unequal in rank to others or ranked inconsistently with other groups and descriptions. All members then met and discussed their choices.

Hourly meetings continued to be scheduled at times when the whole group was available until a final consensus was achieved that the tool was ready for use.

## 3. Results

Six academics with expertise in rural Australian food environments were identified to form the expert group for consensus. All experts held PhD qualifications. Other qualifications included a Bachelor of Nutrition and Dietetics (n = 3), a Bachelor of Health Science (n = 2), and a Bachelor of Science (n = 1). One member was an accredited practising dietitian (APD), and another two held advanced APD qualifications with Dietitians Australia [29]. Experience ranged from 5 to 28 years. Two experts covered the New England region of New South Wales (NSW), with others representing the mid–north coast of NSW, Western Victoria, Tasmania, and regional Western Australia.

### 3.1. Results from Stage 1: Identification of Rural Food Outlets of Interest

Outlets derived from the literature initially presented to the expert group can be seen in Table 2. Outlets that were added from either the Yellow/White Pages [25] or documented from expert group discussions as either standalone or to be included in an existing category were canteens, community gardens, farmgate suppliers (non-commercial), farmgate suppliers (commercial), food trucks/vans, pizza shops, roadhouses, vending machines, and sport, art, and racing venues.

### 3.2. Results from Stage 2: Development and Modification of Outlet Descriptions

Thirteen categories which included 40 food outlets were described (see Supplementary Materials S1) and gained consensus from the group that the outlets and matching descriptions were comparable to their area. Eight combinations of surveys and online meetings were required to gain consensus for the list of outlets and matching descriptions and took place over a period of approximately 2 months (August to October 2022).

### 3.3. Results from Stage 3: Instructions for Use

The drafted *instructions for use* that were developed covered the scope of remoteness, which included MM3–5 areas according to the Modified Monash Model [17]. Other key instructions related to core and discretionary foods, which were defined according to the Australian Guide to Healthy Eating [30], mixed ranges of food and beverages, potential methods of ground truthing, and sizes of establishments, as provided by the Australian Government [28]. Additionally, instructions were developed describing how to remove alcohol from the scoring if this was not the intent or scope of the user.

### 3.4. Results from Stage 4: Providing a Healthiness Score to Included Outlets with Descriptions

Two rounds of individual scoring were required to show where consensus was able to be gained and where disagreement was not able to be solved. Further modifications to the drafted food outlet list and accompanying descriptions were required and are shown in Supplementary Materials S2.

Hot chicken and chips shop; fish and chips shop; wholesale/food cooperative; sport, art, and racing venues; vending machines and convenience stores were discussed and not included in the draft version due to their similarity to other outlet types provided or limited exposure in the population. A total of 43 outlets were drafted for final inclusion and scoring (see Supplementary Materials S2).

### 3.5. Results from Stage 5: Consistency and Consensus

The expert group was provided with the draft food outlet-scoring tool and the scores from Stage 4. They were also provided with visual summaries of the relative placements of each type of outlet for their consideration. Each expert prepared for an online group meeting to discuss any remaining concerns with the tool and to provide a robust discussion regarding final scoring options.

All outlets described were compared to the original scoring system documented by Moayyed et al. [24] to ensure consistency with pre-existing literature. Additionally, all food outlets described were compared to each other. The final version achieved consensus in the online meeting. Following the group meeting, a refined copy of the agreed tool was sent to each group member so they could consider, individually, whether any further changes were necessary. All group members provided written consent that they were satisfied with the final version, including the instructions for use, presented in Supplementary Materials S3. It contains 12 categories of food outlets and 35 individual outlets listed. Scores range from +10 (local produce stall, community gardens, and non-commercial farmgate suppliers) to −10 (bottle shop or liquor store). Retail outlet scores range based on whether the majority of foods offered are consistent with core or non-core foods, as defined in national health guidelines [30].

## 4. Discussion

In this study, we have presented the development of an Australian-first, healthiness-scoring tool for food outlets that is relevant to Australian rural food environments, developed by nutrition experts who are—and have been—embedded in rural Australian communities. This rural food outlet-scoring tool was built on the previous work of Needham et al. and Moayyed et al. [23,24] and adapted through a modified Delphi technique by a group of rural nutrition experts. The final food outlet-scoring tool includes a list of food outlets commonly found in rural communities categorised within MM3–5 [17], descriptions of each outlet, an accompanying healthiness score, and instructions for use. The tool differs from previous tools through the nuanced descriptions of each outlet type to account for a range of rural contexts. Additionally, descriptions are based on core and discretionary (non-core) foods and beverages as per national guidelines, and the extent of these foods offered within the outlet to better reflect the mixed-outlet models common in rural Australian communities. The healthiness score ranges from −10 (discretionary options offered), through 0 (mixed core and discretionary options), to +10 (core food options) and accounts for the inclusion or non-inclusion of alcohol, both by itself and as part of a meal to reflect the differences in alcohol availability in rural communities. Additionally, the instructions for use provide guidance on what constitutes a core or discretionary food, how the range may be interpreted, a measure for the size of the outlet, and how to score establishments if alcohol is not within the scope required, which ensures that the diversity of rural Australian food environments is encompassed.

The new rural food environment scoring tool differs significantly in terms of outlet types and descriptions compared with previously developed food-scoring tools in Australia [15,23,24,31]. One notable difference is the inclusion of new retail outlets like roadhouses and canteens, which were previously not accounted for. These unique food outlets are particularly relevant in rural areas where food options are limited, and they can serve as important sources of food (both healthy and unhealthy foods) within a community [32]. Additionally, the updated tool can more accurately assess the healthiness of food outlets with mixed purposes, such as general stores that also sell alcohol and/or takeaway meals. This improvement addresses the limitations of previous food-scoring tools, particularly in underestimating alcohol sources in rural communities.

Another enhancement in the new scoring tool that is relevant to the rural context is the distinction made between specific types of food businesses that sell core foods in rural regions. For instance, the tool ensures that fishmongers, poultry shops, and butchers that predominantly sell core foods are distinguished from chicken takeaways or fried fish and chip shops, enabling the identification of healthy and unhealthy retail outlets more accurately, with the healthiness categorisation being based on national guidelines [30]. Similarly, differentiating bread shops and bakeries is crucial in certain MM3–5 areas in Australia, although they have been measured in the same group in other tools [31]. For instance, a store that primarily focuses on selling bread as a core food should not be grouped together with a bakery that predominantly sells cakes and pies. The inclusion of whole foods stores, including community food co-ops, is another important addition, as these outlets can serve as significant sources of core foods in a community while also functioning as social enterprises [33,34]. Lastly, the updated tool incorporates expanded categories such as local produce stalls, community gardens, and non-commercial and commercial farmgate suppliers. These categories primarily involve the sale of core foods and are particularly important sources of foods in regions with abundant food production and strong, localised food supply chains, such as Tasmania and southwest Western Australia [35,36]. By including these new categories, the scoring tool ensures the accurate representation of healthy food outlets that can positively impact population diets in some rural regions.

The tool also offers a wider range of options when scoring restaurants and cafes to more sensitively measure those outlets that sell predominantly core foods, non-core foods, or a mixture of both. This will enhance the tool’s sensitivity in matching outlets with an appropriate or evolving healthiness rating. In addition to core food outlets, the new scoring tool also recognizes the importance of non-core food outlets in rural areas. For instance, gourmet food shops, wineries, cheese stores, and similar establishments predominantly catering to tourists are now included [37]. Capturing these outlets as sources of food within a community is crucial due to their impact on local food availability. The extent to which these contribute to the diet of local residents should be investigated in future research. Expanding the evaluation options for pubs, hotels, and clubs was also critical to better reflect the food environment in rural regions [15]. The updated tool provides greater sensitivity to account for the variety of foods offered, wherein the authors consider that a larger range generally indicates that healthier options are more able to be chosen from a menu. For instance, some pubs may only have vending machines or offer pre-packaged foods over the bar with limited or no core options, resulting in a low score. On the other hand, clubs with a broader range of food choices are likely to include at least some core options such as salads and meat/vegetable dishes alongside non-core fried options, leading to a higher score reflecting their improved healthiness rating.

While this tool has the potential to be a useful resource to understand rural community food environments, it needs to be validated across various Australian regions to understand its accuracy, usefulness, and ability to capture the place-based nuances across locations. For example, the aforementioned ‘combination outlets’ are commonplace in many rural areas. It also needs to be applied within various settings, such as in towns of differing sizes, varied community food environments, and supermarket types, to test its robustness. It may need to be assessed in conjunction with retailer outlets’ perceptions of demand for healthy foods. These may potentially be perceived to decrease profitability due to a reduction in sales or an increase in loss because of perishability [38,39].

This tool is suitable for application within food outlets and across community food environments from small rural towns to large rural towns. In practice, the tool could be utilised by public health nutritionists or dietitians, environmental officers, and/or local councils to understand and benchmark their regional food environment. This evidence could inform practical advice as to where clients and residents can source nutritious foods in the local area, or advocacy activities, such as advocating for local government zoning changes to favour more healthy outlet types, such as fruit and vegetable shops or whole foods and grain stores [40]. Specific outlets can be ranked according to healthiness and mapped in proximity to areas of interest [23]. This information could provide evidence for the petition of health professionals and residents for changes to state government planning laws to prevent the further proliferation of unhealthy outlets around such settings where high-priority or vulnerable populations frequent [41]. Additionally, it could be used to determine locations in which the food supply chain requires further resourcing. In Australia, the distances between communities, as well as variations in temperature and transportation options, impact the supply chain, reducing access to perishable foods [42]. Potential advocacy areas could include Planning and Development Regulation amendments to include ‘public health’ as a planning consideration, particularly in lower socio-economic locations, and to indicate which locations require freighting support and possible increased capacity building [41,42]. Built environment professionals could also utilise this tool to map outlet density, type, and proximity in relation to transport modes. This could inform future commercial planning of residential areas to ensure that healthy food is accessible by public and active transport, which not only benefits community members [43] but also includes local businesses located on transport routes [44]. The mapping of community gardens and non-commercial farmgate availability offers further opportunities to provide personalised place-based information to residents about where to source healthy food. The scant existing evidence suggests that interventions mapped to food environments have successfully increased vegetable intake; however, this is an area requiring further research [45].

Future research needs to consider that tools developed to measure rural environments should be iterative and measured in multiple locations. Previous research understanding differences in neighbourhood characteristics across different remoteness areas of the Modified Monash Model has shown that areas from MM3–5 are relatively comparable to communities across Australia [46]. Further data are needed to compare the different food environment measures, along with comparing this tool with internal food environment measures to ensure accurate characterisation of the healthiness of different food environments. Previous research on food environment scoring has shown large variability between the different measures in rural Australia [47]. Internationally, a review of the literature between 2007 and 2015 identified 432 studies that measured the food environment, but further detail is required as to which tools were specifically designed for rural areas [48]. Data will need to be collected within outlets over a series of years and will need to be an area for future research.

The strengths of this study include the involvement of qualified rural nutrition experts with a diverse geographic representation, and that the scoring of the retail outlets is based on national guidelines. The tool was developed using a systematic and transparent process utilising various stages of iterative feedback. This process may be producible by experts in other international rural contexts requiring similar adaptations to be made. However, an inherent limitation of the Delphi technique is that the results are opinion-based. However, given the limited data available on rural food environments, expert opinion and consensus were considered an appropriate avenue for the exploration of this topic. The expertise and experience of the respondents further support this choice of methodology [18]. While measures were taken to ensure the anonymity of responses where possible, certain aspects were conducted online. This could have led to some members dominating the discussion, potentially introducing social desirability bias or groupthink [21]. Further, not all group members were able to attend every online meeting. To overcome this limitation, all sessions were recorded and made available to absent members, who were asked to send their contribution for that session in written format. All members were present for Stage 5 of the study, and all contributed to the final reflection on accuracy. It is also possible that the final tool developed may not have captured all available food outlets. The tool is considered comprehensive for the areas represented by the expert panel; however, the panel did not represent all states and territories in Australia (such as the Northern Territory and Queensland). It is also possible that experts meeting the inclusion criteria for the panel were not identified. Finally, the tool does not incorporate remote communities (MM6–7) due to inherent differences in the food environment in these communities, but this remains an important area for future research.

## 5. Conclusions

The rural food environment scoring tool represents a novel method that may increase the accuracy of describing and mapping rural food environments. It may provide a foundation for standardised food mapping in the Australian rural context. Future research could lead to the development of a tool for the remote Australian context and provide the outline of a process to develop or adapt similar tools internationally. Subsequent research should include a synthesis of international tools and their development so that a consistent process can be identified for areas that currently do not have a way of measuring local food environments. Additionally, areas designed as MM3–5 in Australia may be able to investigate the impact of rural food environments on the health of local populations to increase the evidence available on the impact of healthy food environments on rural policymakers. Such data can support local governments in making informed policy decisions regarding how to support improvements for obtaining equitable access to healthy foods.

## Figures and Tables

**Table 1:** First form of the survey aimed to capture a level of agreement regarding the type and descriptions of food outlets included in the tool.

Part	Source of Food Outlet	Instructions to Group of Experts
A: Food outlets with existing descriptions	Descriptions provided from literature: Needham et al. [23]	Experts were asked to indicate their level of agreement with the existing proposed description(s) specific to a rural context (strongly agree/agree/neutral/ disagree/strongly disagree). Modifications could be suggested if desired. Sub-categories of food retail outlets could also be proposed, and a suggested description was provided.
B: Food outlets without an existing description	From Moayyed et al. [24]	Participants were asked to propose a description specific to a rural context. Descriptions from non-literature sources (e.g., government websites, Wikipedia) were provided as a base reference for editing.
C: Food outlets that may fit within an existing category	From Yellow/White Pages [25] and expert group	Participants were asked to indicate whether: (a) the outlet appropriately fitted within an existing category (and to nominate this category); (b) the outlet should not be included in the tool; or (c) the outlet should be a ‘stand-alone’ category (and to propose a description for a rural context). Existing similar categories were provided for consideration.

**Table 2:** Food outlet types provided to expert consensus group for consideration from the literature.

Food Outlet Type	Moayyed et al. (2017) [24] Healthiness Score	Needham et al. (2020) [23] Healthiness Score	Pre-Existing Description from Needham et al. [23]
Fruiterer and greengrocer	10	10	☑
Local produce stall	10	Not included	⊠
Fish shop	10	9	☑
Poultry shop	9	9	☑
Butchery	9	9	☑
Farmer’s market	8	Not included	⊠
Wholesaler/food coop	8	Not included	⊠
Major supermarket	5	5	☑
Minor supermarket	5	5	☑
Specialty food store (core)	5	5	☑
Restaurant/café (franchise)	0	0	☑
Restaurant/café (local)	0	0	☑
Sandwich shop	5	5	☑
Salad/sushi bar	Not included	5	☑
Delicatessen	0	0	☑
Bakery/cake shop	0	0	☑
Pharmacy	−5	Not included	⊠
Others	−5	Not included	⊠
Convenience store	−5	Not included	⊠
Specialty food (extra foods)	−8	−8	☑
Pub	−8	−5	☑
Takeaway (local)	−8	−8	☑
Takeaway (franchise)	−10	−10	☑
Service station convenience	−10	Not included	⊠
Liquor-selling shop	−10	Not included	⊠
General store	Not included	−5	☑

☑ = item is included in pre-existing descriptions, ⊠ = item is not included in pre-existing descriptions.

## Data Availability

Data can be made available upon reasonable written request to the corresponding author.

## Supplementary Materials

The following supporting information can be downloaded at: https://www.mdpi.com/article/10.3390/nu15214660/s1, Supplementary Materials S1: Updated food outlets list with descriptions that was completed in Stage 2; Supplementary Materials S2: Draft food outlet-scoring tool (results from Stage 4); Supplementary Materials S3: Rural food outlet-scoring tool with instructions for use.
