# Supplementary material for: Development of a Scoring Tool for Australian Rural Food Retail Environments
Source: Nutrients. 2023 Nov 3;15(21):4660. doi: 10.3390/nu15214660 (PMC10648429; doi:10.3390/nu15214660)
Supplement: Supplementary file 1 [file nutrients-15-04660-s001.zip › Supplementary materials S1.pdf]

Supplementary material 1. Updated food outlets list with descriptions that was completed in Stage 2. Modifications to text were added by consensus by the expert group to describe outlets in their own area.

Food Outlet Type	Description
**Supermarkets and Grocery Stores**	
Major supermarket	Mainly engaged in the sale of groceries (fresh foods, canned and packaged foods, dry goods) of non-specialised (conventional) food lines. May contain a butcher or baker. Usually have 5 or more checkouts and a floor area over 1000 square metres. i.e., Woolworths, Coles, ALDI.
Minor supermarket	Mainly engaged in the sale of groceries (fresh foods, canned and packaged foods, dry goods) of non-specialised (conventional) food lines. May contain a butcher or baker. Usually have 4 or fewer checkouts and a floor area under 1000 square metres. e.g., independent grocer or supermarket.
Major or minor supermarket with alcohol	Mainly engaged in the sale of groceries (fresh foods, canned and packaged foods, dry goods) of non-specialised (conventional) food lines. May contain a butcher or baker. Sells alcohol (contained within the premises). e.g., ALDI plus liquor; IGA plus liquor.
Supermarket - unknown	Mainly engaged in the sale of groceries (fresh foods, canned and packaged foods, dry goods) of non-specialised (conventional) food lines. Unknown number of checkouts or floor area.
**Food and/or General Stores**	
Food and/or general store	Mainly engaged in the sale of a limited line of groceries; generally includes milk, bread and canned and packaged foods. Range is more limited than a minor supermarket and there may be wide variability in the proportion of core and non-core foods. Foods may vary seasonally.
Food and/or general store with alcohol and/or takeaway food	Mainly engaged in the sale of a limited line of groceries; generally includes milk, bread and canned and packaged foods. Range is more limited than a minor supermarket and there may be wide variability in the proportion of core and non-core foods. Foods may vary seasonally. Also sells alcohol (contained within the premises) and/or takeaway foods e.g., burgers, fried foods.
Food and/or general store - unknown	Mainly engaged in the sale of a limited line of groceries; range of foods available is unknown.
**Butchers and Poultry Shops**	
Butcher and/or poultry shop	Mainly engaged in the sale of fresh or frozen meat and/or poultry; includes wholesale stores with direct-to-public sales.
Poultry shop with cooked and/or discretionary food	Mainly engaged in the sale of fresh or frozen meat and/or poultry; includes wholesale stores with direct-to-public sales. Also sells cooked and/or discretionary food that may be available for takeaway e.g., nuggets, burgers, hot chips
Hot chicken and chips shop	(i.e., cooked food items only) *See 'Take away food'*
Butcher and/or poultry shop - unknown	Mainly engaged in the sale of meat and/or poultry; range of products available is unknown.
Fish and Seafood Shops	

Food Outlet Type	Description
Fish and seafood shop (fishmonger)	Mainly engaged in the sale of fresh or frozen seafood for preparation off premises; includes wholesale stores with direct-to-public sales.
Fish and seafood shop with cooked food	(Mainly?) engaged in the sale of fresh or frozen seafood for preparation off premises; also sells cooked food items for consumption on or off premises. Includes wholesale stores with direct-to-public sales and takeaway stores that provide a range of fresh seafood.
Fish and chip shop	(i.e., cooked food items only) *See 'Take away food'*
Fish and seafood shop - unknown	Mainly engaged in the sale of seafood; range of products available is unknown.
**Bakers**	
Bread shop	Mainly oriented towards bread~~, biscuits, pastries or other flour~~ products, with or without packaging, including traditional and artisan breads. May contain minimal amounts of other non-bread discretionary items, such as pastries or baked goods.
Bakery	Mainly oriented towards discretionary baked goods, such as ~~bread,~~ biscuits, pastries, pies, or other flour products, with or without packaging. Also sells limited bread (or other core) products.
Bread shop or bakery - unknown	Mainly oriented towards bread and/or baked goods; range of core/discretionary products available is unknown.
**Specialty Food Stores – Mixed Core and Discretionary Foods**	
Gourmet food stores/ fine grocer?	Mainly engaged in the sale of specialty packaged or fresh products; contains a mixture of core and discretionary foods (e.g., cured meats, sausage, cheese, pickled vegetables, oils, dips, artisan bread and crackers, olives). May showcase regional foods and provide dine-in options.
Delicatessens?	Mainly engaged in the sale of specialty packaged or fresh core and discretionary products ~~such as~~ cured meats and sausages; may also sell dips, cheeses, bread, and olives. May provide dine-in options.
Gourmet food store - unknown	Mainly engaged in the sale of specialty packaged or fresh products; range of core/discretionary products available is unknown.
**Specialty Food Stores – Core Foods**	
Wholefoods and grain stores	Mainly engaged in the sale of ~~a limited line of~~ specialty wholefoods and grains ~~specialised food, such as~~ (e.g., dried lentils, seeds, nuts, dried fruits); items may be organic, packaged or unpackaged, and ~~a particular gourmet food that~~ can be defined under core food.
Cheese shop	Mainly engaged in the sale of specialty cheeses and other dairy products
Specialty food store (core foods) - unknown	Mainly engaged in the sale of specialty core foods – range of products available is unknown.
**Specialty Food Stores – Discretionary Foods**	
Specialty food store – discretionary foods	Mainly engaged in the sale of specialty discretionary foods and beverages (e.g., ~~such as~~ ice-creams, donuts, waffles, cakes, confectionery, chocolate, etc.).
Cake and pastry shop	Mainly engaged in the sale of cakes, pastries, or other discretionary flour products. Does not sell bread. *See 'bakery' for outlets that sell bread products.*

Food Outlet Type	Description
Specialty food store (discretionary foods) - unknown	Mainly engaged in the sale of specialty discretionary foods – range of core/discretionary products available is unknown.
**Fruiterers & Greengrocers**	
Fruit and vegetable shop	Mainly engaged in the sale of fresh fruit and vegetables; including wholesale stores with direct to public sales. May contain a limited range of other core and discretionary foods, such as juices, pasta, sauces, nuts, crackers, and confectionery.
Fruit and vegetable shop - unknown	Mainly engaged in the sale of fresh fruit and vegetables; range of other core and discretionary foods is unknown (e.g., juices, pasta, sauces, nuts, crackers and confectionery).
**Cafes & Restaurants**	
Café/restaurant – discretionary foods	Mainly engaged in the preparation and sale of discretionary meals/snacks for consumption on the premises; table service provided; may sell alcohol with food; may provide takeaway services (but is not a fast-food outlet). *Provide examples? For example, coffee shop with a wide range of cakes and slices available, or restaurant with mainly high fat/salt/sugar (such as battered or fried) options available.*
Café/restaurant –core foods	Mainly engaged in the preparation and sale of core food meals/snacks for consumption on the premises; table service provided; may sell alcohol with food; may provide takeaway services (but is not a fast-food outlet). *Provide examples? For example, café or restaurant with mainly salad/soup/sandwich options*
Café/restaurant – mixed or unknown	Mainly engaged in the preparation and sale of a mixture of discretionary and core food meals/snacks (OR range of food options is unknown) for consumption on the premises; table service provided; may sell alcohol with food; may provide takeaway services (but is not a fast-food outlet). *Provide examples?*
**Take Away/Fast Food**	
Take away – discretionary foods	Mainly engaged in the preparation and sale of discretionary meals/snacks (e.g., kebab, fish & chips, chicken & chips, burgers, pizzas). Food is ready for immediate consumption; table service not typically provided; meals can be eaten on site, taken away or delivered~~; shop is not a franchise~~. Excludes donuts, drinks, and ice-cream (see *'Specialty food store – discretionary'*). ~~The food shop is a franchise/chain store with food being sold in specialised packaging.~~
Take away —core foods	Mainly engaged in the preparation and sale of core food meals/snacks (e.g., choice of salads, wraps, sandwiches). Food is ready for immediate consumption; table service not typically provided; meals can be eaten on site, taken away or delivered~~; shop is not a franchise~~. *Include Mexican? Sushi?*
Take away - mixed or unknown	Mainly engaged in the preparation and sale of a mixture of discretionary and core food meals/snacks (e.g., burgers, fries, pizzas, fried foods, sandwiches, choice of salads, sushi; OR range of food options is unknown). Food is ready for immediate consumption; table service not typically

Food Outlet Type	Description
	provided; meals can be eaten on site, taken away or delivered. Excludes donuts, drinks, and ice-cream (see *'Specialty food store – discretionary'*).
**Pubs, Clubs & Hotels**	
Pub, club, hotel – large range	Venue that predominantly serves alcohol, with a large range of food options available, including individually designated restaurants/venues within the establishment.
Pub, club, hotel – limited range	Venue that predominantly serves alcohol, with a limited range of food options available. For example, those offering counter meal-type menus.
Pub, club, hotel – packaged foods	Venue that predominantly serves alcohol, but has only pre-packaged, shelf-stable or vending machine-type foods available as food options.
Pub, club, hotel - unknown	Venue that predominantly serves alcohol; range of food options available is unknown.
**Bottle Shops & Liquor Stores**	
Bottle shop or liquor store	Mainly engaged in the sale of alcoholic beverages to the public for consumption off premises. Sale of alcohol is separate if co-located (e.g., separate to supermarket or general store); may offer home delivery and/or mail order.
NB Red text indicates modification, addition or deletion to existing description by expert group<br>NB Blue text indicates addition of a new category or sub-category of food outlet by expert group	
