# Supplementary material for: Development of a Scoring Tool for Australian Rural Food Retail Environments
Source: Nutrients. 2023 Nov 3;15(21):4660. doi: 10.3390/nu15214660 (PMC10648429; doi:10.3390/nu15214660)
Supplement: Supplementary file 1 [file nutrients-15-04660-s001.zip › Supplementary materials S3.pdf]

Supplementary Material S3: Rural food outlet scoring tool with instructions for use

Instructions for Use:

The following tool is designed to be flexible for the number of different contexts that may be found in rural Australian food environments. Please note, this does not cover REMOTE communities. In this tool, we designate that this tool may be suitable for areas categorised as MM3 (Large rural towns) to MM5 (Small rural towns) according to the Modified Monash Model [1].

Information is provided below that may be useful when considering the categories within the scoring tool.

Core and discretionary foods. This tool uses definitions for core and discretionary foods as provided by the Australian Guide to Healthy Eating [2]. Categories that describe food sources of core and discretionary foods are directly linked to their healthiness score.

Core foods are those that are listed in the five food groups): [https://www.eatforhealth.gov.au/food-essentials/five-food-groups]

- Fruit
- Grain (cereal) foods, mostly wholegrain and / or high cereal fibre varieties
- Lean Meat and poultry, fish, eggs, tofu, nuts and seeds and legumes/beans
- Milk, yoghurt, cheese and / or their alternatives (mostly reduced fat)
- Vegetables and Legumes / Beans

Please note that no differentiation is given here for core foods that are either **fresh or frozen –** both are considered to score equally.

Discretionary (or non-core) foods are those with higher added sugars, higher fat, higher fat and added sugar or those with high alcohol: https://www.eatforhealth.gov.au/food-essentials/discretionary-food-and-drink-choices.

Venues that are described as providing **Mainly Core** or **Mainly Discretionary** foods recognise that very few venues sell only one category of food. Instead, there may be a predominance or greater availability of either core or discretionary as the main purpose of the establishment.

Venues may also be described as having **Mixed or Unknown availability** of core and discretionary foods. These options should be chosen if ground truthing is not available or there is a relatively equal mix of core and discretionary foods on offer.

Extensive and limited range: Some venues may be described as having an extensive or limited range of foods available. This refers to the number of items available, rather than the type (core or discretionary). A limited range implies limited choice available.

Seasonal foods: some venues may stock a greater range of seasonal produce or operate at limited times of the year with seasonal produce. This is described where appropriate.

Ground truthing refers to the process of when a researcher makes themselves familiar with the establishment or venue in question. This may be through personal visits, or in the case with more distance places, uses external sources such as google maps or venue websites to determine the type or range of foods and beverages sold (e.g. through online menus or range of stock advertised).

The inclusion of **alcohol** in the tool may not be of interest to the user. If the sale of alcohol is not within your scope, score establishments such as pubs, clubs and hotels according to the closest restaurant/café descriptor.

If desired, a venue or establishment can also be **weighted according to its size**. The size of the establishment/venue can be categorised according to information adapted from the definitions for small businesses in Australia [3] to refer to individual venues:

Micro venue/establishment/Limited access: A business that employs between 1-4 people, including those that are sole proprietorships and partnerships without employees. (Score 1)

Small venue/establishment /Moderate access: A business that employs between 5-19 employees (Score 2)

Medium to Large venue/establishment /High access: A business that employs 20-+employees (Score 3)

References:

1. Australian Government Department of Health. Modified Monash Model. 2020 [cited 2020 7 Feb 2020]; Available from: https://www.health.gov.au/health-workforce/health-workforce-classifications/modified-monash-model.

2. National Health and Medical Research Council. Australian Guide to Healthy Eating. 2013 01 May 2017 [cited 2021 15 Oct 2021]; Available from: https://www.eatforhealth.gov.au/guidelines/australian-guide-healthy-eating.

3. Parliament of Australia. Definitions and data sources for small business in Australia: a quick guide. Research paper series, 2015-16 2015 [cited 2022 08 Dec 2022]; Available from: https://www.aph.gov.au/about_parliament/parliamentary_departments/parliamentary_library/pubs/rp/rp1516/quick_guides/data.

Table S1: MM3-5 Rural food outlet scoring tool

Food Outlet	Description	Score
**Supermarkets and Grocery Stores**		
Major supermarket	Mainly engaged in the sale of groceries (fresh foods, canned and packaged foods, dry goods) of non-specialised (conventional) food lines. May contain a butcher or baker. Usually have 5 or more checkouts and a floor area over 1000 square metres. i.e., Woolworths, Coles, ALDI.	5
Minor supermarket	Mainly engaged in the sale of groceries (fresh foods, canned and packaged foods, dry goods) of non-specialised (conventional) food lines. May contain a butcher or baker. Usually have 4 or fewer checkouts and a floor area under 1000 square metres. e.g., independent grocer or supermarket.	4
Major or minor supermarket with alcohol	Mainly engaged in the sale of groceries (fresh foods, canned and packaged foods, dry goods) of non-specialised (conventional) food lines. May contain a butcher or baker. Sells alcohol (contained within the premises). e.g., ALDI plus liquor; IGA plus liquor.	2
Supermarket - unknown	Mainly engaged in the sale of groceries (fresh foods, canned and packaged foods, dry goods) of non-specialised (conventional) food lines. Unknown number of checkouts or floor area.	4
**Food and/or General Stores**		
Food and/or general store	Mainly engaged in the sale of a limited line of groceries; generally includes milk, bread and canned and packaged foods. Range is more limited than a minor supermarket and there may be wide variability in the proportion of core and non-core foods. Foods may vary seasonally.	2
Food and/or general store with alcohol and/or takeaway food	Mainly engaged in the sale of a limited line of groceries; generally includes milk, bread and canned and packaged foods. Range is more limited than a minor supermarket and there may be wide variability in the proportion of core and non-core foods. Foods may vary seasonally. Also sells alcohol (contained within the premises) and/or takeaway foods e.g., burgers, fried foods.	-7
Food and/or general store - unknown	Mainly engaged in the sale of a limited line of groceries; range of foods available is unknown.	-3
Service station convenience store	Primarily sells petrol/diesel and pre-prepared foods-to-go, snacks and take away foods. It may also sell frozen goods, dairy foods, bakery items, beverages and a range of non-food items.	-7

Table S1: MM3-5 Rural food outlet scoring tool

Food Outlet	Description	Score
Roadhouse	Restaurant/cafe based at a service station/petrol station that provides cooked meals for travellers with a sit-down option that includes main meals and/or take away food options; meal options typically over 12-24 hours.	-6
Canteens	An outlet that is mainly engaged in the preparation and sale of mainly non-core meals/snacks for consumption of people within an institution like a school, camp, sporting facilities or workplace and is often volunteer run.	-5
**Butchers and Poultry Shops**		
Butcher and/or poultry shop	Mainly engaged in the sale of fresh or frozen meat and/or poultry; includes wholesale stores with direct-to-public sales.	9
Poultry shop with cooked and/or discretionary food	Mainly engaged in the sale of fresh or frozen meat and/or poultry; includes wholesale stores with direct-to-public sales. Also sells cooked and/or discretionary food that may be available for takeaway e.g., nuggets, burgers, hot chips.	0
Butcher and/or poultry shop - unknown	Mainly engaged in the sale of meat and/or poultry; range of products available is unknown.	4
**Fish and Seafood Shops**		
Fish and seafood shop (fishmonger)	Mainly engaged in the sale of fresh or frozen seafood for preparation off premises; includes wholesale stores with direct-to-public sales.	9
Fish and seafood shop with cooked food	Mainly engaged in the sale of fresh or frozen seafood for preparation off premises; also sells cooked food items for consumption on or off premises. Includes wholesale stores with direct-to-public sales and takeaway stores that provide a range of fresh seafood.	0
Fish and seafood shop - unknown	Mainly engaged in the sale of seafood; range of products available is unknown.	4
**Bakers**		
Bread shop	Mainly oriented towards bread products, with or without packaging, including traditional and artisan breads. May contain minimal amounts of other non-bread discretionary items, such as pastries or baked goods.	9
Bakery	Mainly oriented towards discretionary baked goods, such as biscuits, pastries, pies, or other flour products, with or without packaging. Also sells limited bread (or other core) products.	-5

Table S1: MM3-5 Rural food outlet scoring tool

Food Outlet	Description	Score
Bread shop or bakery - unknown	Mainly oriented towards bread and/or baked goods; range of core/discretionary products available is unknown.	-2
**Specialty Food Stores – Mixed Core and Discretionary Foods**		
Gourmet food stores and delicatessens	Mainly engaged in the sale of specialty packaged or fresh products; contains a mixture of core and discretionary foods (e.g., cured meats, sausage, cheese, pickled vegetables, oils, dips, artisan bread and crackers, olives). May showcase regional foods and provide dine-in options.	0
Gourmet food stores and delicatessens with alcohol	May be part of a vineyard cellar door with dine-in or takeaway food product options.	-5
Gourmet food store - unknown	Mainly engaged in the sale of specialty packaged or fresh products; range of core/discretionary products available is unknown.	-3
**Specialty Core Food Stores**		
Wholefoods and grain stores	Mainly engaged in the sale of specialty wholefoods and grains (e.g., dried lentils, seeds, nuts, dried fruits); items may be organic, packaged or unpackaged, and can be defined under core food.	9
Cheese shop	Mainly engaged in the sale of specialty cheeses and other dairy products	3
Specialty core food store - unknown	Mainly engaged in the sale of specialty core foods – range of products available is unknown.	5
**Specialty Discretionary Food Stores**		
Specialty discretionary food store	Mainly engaged in the sale of specialty discretionary foods and beverages (e.g., ice-creams, donuts, waffles, cakes, confectionery, chocolate, etc.).	-7
Cake and pastry shop	Mainly engaged in the sale of cakes, pastries, or other discretionary flour products. Does not sell bread. *See 'bakery' for outlets that sell bread products.*	-9
Specialty discretionary food store - unknown	Mainly engaged in the sale of specialty discretionary foods – range of core/discretionary products available is unknown.	-7

Table S1: MM3-5 Rural food outlet scoring tool

Food Outlet	Description	Score
**Fruiterers & Greengrocers**		
Fruit and vegetable shop	Mainly engaged in the sale of fresh fruit and vegetables, including wholesale stores with direct to public sales. May contain a limited range of other core and discretionary foods, such as juices, pasta, sauces, nuts, crackers, and confectionery.	9
Fruit and vegetable shop - unknown	Mainly engaged in the sale of fresh fruit and vegetables; range of other core and discretionary foods is unknown (e.g., juices, pasta, sauces, nuts, crackers and confectionery).	8
Local produce stall, community gardens and non-commercial farmgate suppliers	Mainly engaged in the sale of limited numbers of locally grown core foods such as fruit, vegetables, eggs, honey or home-made products to members of a particular community, often at a reduced price.	10
Farmers market and commercial farmgate suppliers	Mainly engaged in the sale of core foods eggs, meat, dairy, honey, fruit and vegetables grown locally. May also be engaged in the sale of non-core foods such as jams and spreads	9
**Cafes & Restaurants**		
Café/restaurant – discretionary foods	Mainly engaged in the preparation and sale of discretionary meals/snacks for consumption on the premises; table service provided; may sell alcohol with food; may provide takeaway services (but is not a fast-food outlet). *For example, coffee shop with a wide range of cakes and slices available, or restaurant with mainly high fat/salt/sugar (such as battered or fried) options available.*	-7
Café/restaurant – core foods	Mainly engaged in the preparation and sale of core food meals/snacks for consumption on the premises; table service provided; may sell alcohol with food; may provide takeaway services (but is not a fast-food outlet). *For example, café or restaurant with mainly salad/soup/sandwich options.*	5
Café/restaurant – mixed or unknown	Mainly engaged in the preparation and sale of a mixture of discretionary and core food meals/snacks (OR range of food options is unknown) for consumption on the premises; table service provided; may sell alcohol with food; may provide takeaway services (but is not a fast-food outlet).	0

Table S1: MM3-5 Rural food outlet scoring tool

Food Outlet	Description	Score
**Take Away/Fast Food**		
Take away – discretionary foods	Mainly engaged in the preparation and sale of discretionary meals/snacks (e.g., kebab, fish & chips, chicken & chips, burgers, pizzas). Food is ready for immediate consumption; table service not typically provided; meals may be eaten on site, taken away or delivered. May include pop-ups, food trucks and vans. Excludes donuts, drinks, and ice-cream (see *'Specialty food store – discretionary'*).	-9
Take away – core foods	Mainly engaged in the preparation and sale of core food meals/snacks (e.g., choice of salads, wraps, sandwiches). Food is ready for immediate consumption; table service not typically provided; meals may be eaten on site, taken away or delivered. May include pop-ups, food trucks and vans.	5
Take away - mixed or unknown	Mainly engaged in the preparation and sale of a mixture of discretionary and core food meals/snacks (e.g., burgers, fries, pizzas, fried foods, sandwiches, choice of salads, sushi; OR range of food options is unknown). Food is ready for immediate consumption; table service not typically provided; meals may be eaten on site, taken away or delivered. May include pop-ups, food trucks and vans. Excludes donuts, drinks, and ice-cream (see *'Specialty food store – discretionary'*).	-3
**Pubs, Clubs & Hotels**		
Pub, club, hotel – large range	Venue that predominantly serves alcohol, with a large range of food options available, including individually designated restaurants/venues within the establishment.	-5
Pub, club, hotel – limited range	Venue that predominantly serves alcohol, with a limited range of food options available. For example, those offering counter meal-type menus.	-6
Pub, club, hotel – packaged foods	Venue that predominantly serves alcohol, but has only pre-packaged, shelf-stable or vending machine-type foods available as food options.	-10
Pub, club, hotel - unknown	Venue that predominantly serves alcohol; range of food options available is unknown.	-8
Bottle shop or liquor store	Mainly engaged in the sale of alcoholic beverages to the public for consumption off premises. Sale of alcohol is separate if co-located (e.g., separate to supermarket or general store); may offer home delivery and/or mail order.	-10
